# Prediction of novel synthetic pathways for the production of desired chemicals

**DOI:** 10.1186/1752-0509-4-35

**Published:** 2010-03-28

**Authors:** Ayoun Cho, Hongseok Yun, Jin Hwan Park, Sang Yup Lee, Sunwon Park

**Affiliations:** 1 Department of Chemical & Biomolecular Engineering (BK21 program), KAIST, Daejeon, South Korea; 2 Bioinformatics Research Center, KAIST, Daejeon, South Korea; 3 Center for Systems and Synthetic Biotechnology, Institute for the BioCentury, Daejeon, KAIST, South Korea

## Abstract

**Background:**

There have been several methods developed for the prediction of synthetic metabolic pathways leading to the production of desired chemicals. In these approaches, novel pathways were predicted based on chemical structure changes, enzymatic information, and/or reaction mechanisms, but the approaches generating a huge number of predicted results are difficult to be applied to real experiments. Also, some of these methods focus on specific pathways, and thus are limited to expansion to the whole metabolism.

**Results:**

In the present study, we propose a system framework employing a retrosynthesis model with a prioritization scoring algorithm. This new strategy allows deducing the novel promising pathways for the synthesis of a desired chemical together with information on enzymes involved based on structural changes and reaction mechanisms present in the system database. The prioritization scoring algorithm employing Tanimoto coefficient and group contribution method allows examination of structurally qualified pathways to recognize which pathway is more appropriate. In addition, new concepts of binding site covalence, estimation of pathway distance and organism specificity were taken into account to identify the best synthetic pathway. Parameters of these factors can be evolutionarily optimized when a newly proven synthetic pathway is registered. As the proofs of concept, the novel synthetic pathways for the production of isobutanol, 3-hydroxypropionate, and butyryl-CoA were predicted. The prediction shows a high reliability, in which experimentally verified synthetic pathways were listed within the top 0.089% of the identified pathway candidates.

**Conclusions:**

It is expected that the system framework developed in this study would be useful for the *in silico *design of novel metabolic pathways to be employed for the efficient production of chemicals, fuels and materials.

## Background

In the past few decades, various systematic methods have been developed for the prediction of synthetic metabolic pathways for the production of chemicals by employing microorganisms [1-15]. These methods can be classified by whether the approach is based on chemical structural changes, enzymatic information, and/or reaction mechanisms. The method based on chemical structural changes is applied to reconstruct the network which represents the relationship among the biochemical compounds using the structure-based homology analysis [1-4]. This method generates a variety of novel pathways, but prediction to specify the enzymes is difficult. Enzymatic information-based approach focuses on combination of gene knock outs and additions of pathways existing in different organisms [5,6]. This method is practical to use, but predictions are limited to the synthesis of currently known biochemical compounds. Reaction mechanisms-based approach identifies product candidates that can be driven from a predetermined substrate using a knowledge-based expert system [7-10]. This method predicts novel pathways and compounds according to the accumulated knowledge and rules, but it is limited to identifying biodegradation pathways.

To overcome the disadvantages of the aforementioned methods, the pathway prediction systems were established by combining the given reaction mechanisms and the starting and target compounds [11-13]. These approaches can generate novel compounds and reactions with proposed enzyme candidates. However, the starting and target compounds should be set as known compounds, and thus this method is difficult to be applied to the prediction of a synthetic pathway for a novel compound of interest. A retrosynthesis model, which is a functional group-based synthesis method towards a target compound, has been applied to search desired target chemicals [14]. However, the previous studies provided a huge set of predicted pathways, rather than suggesting more favorable pathways to achieve a goal of efficiently producing a desired chemical. In this study, a system framework was developed to suggest promising enzyme candidates to synthesize desired chemicals based on combined information on chemical structural changes, enzyme characteristics, and reaction mechanisms. The proposed system framework identifies structurally qualified enzymes for the synthesis of predetermined target chemicals and then ranks the enzymes via a prioritization scoring algorithm. Recently, a nice scoring technique to identify preferred pathways by using an automatic design approach for the metabolic pathways has been suggested [15]; a scoring algorithm was developed for identifying a possible route from a considered host organism. However, this approach cannot be applied to the novel pathways which are not present in the database. Thus, a new scoring algorithm was developed in this paper for the identification of desired novel synthetic pathways. Consequently, the more efficient metabolic pathways for the production of a desired chemical can be proposed.

## Results and Discussion

Using the system framework developed in this study, the novel synthetic pathways for the production of isobutanol, 3-hydroxypropionate (3HP), and butyryl-CoA were predicted. In summary, the steps composed of definition of a target compound, route generation, prioritization, and parameter optimization were taken to predict the novel synthetic pathways. The prediction shows a high reliability, in which experimentally verified pathways for the synthesis of isobutanol, 3HP, and butyryl-CoA belonged to top 0.047%, 0.044%, and 0.089% of all the predicted pathway candidates, respectively.

### Prediction of synthetic pathways of the production of biofuels and evolutionary parameter optimization

**Figure 1:**
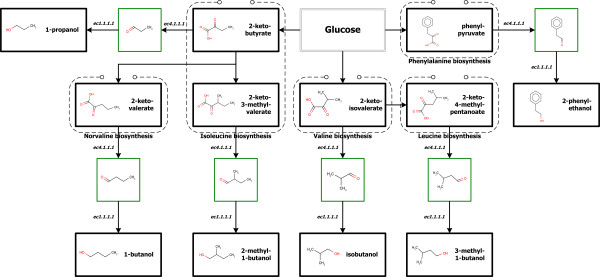
**Six synthetic pathways of higher alcohols**. The synthetic pathways for 1-propanol, 1-butanol, 2-methyl-1-butanol, 3-methyl-1-butanol, isobutanol and 2-phenylethanol with their starting compounds, 2-ketobutyrate, 2-ketovalerate, 2-keto-3-methyl-valerate, 2-keto-4-methyl-pentanoate, 2-ketoisovalerate and phenylpyruvate are shown, respectively.

Recently, Atsumi *et al*. reported novel metabolic engineering strategies for the production of higher alcohols such as 1-propanol, 1-butanol, 2-methy-1-butanol, 3-methy-1-butanol, isobutanol, and 2-phenylethanol in *Escherichia coli *[16]. The six pathways were devised involving two well-known enzymes, 2-keto-acid decarboxylase (KDC, ec4.1.1.1) and alcohol dehydrogenase (ADH, ec1.1.1.1), which is based on the reaction mechanism that 2-keto acids can be converted to aldehydes and then to alcohol. To testify their work, the system framework was employed. The key novel synthetic pathways are presented in Figure 1.

**Figure 2:**
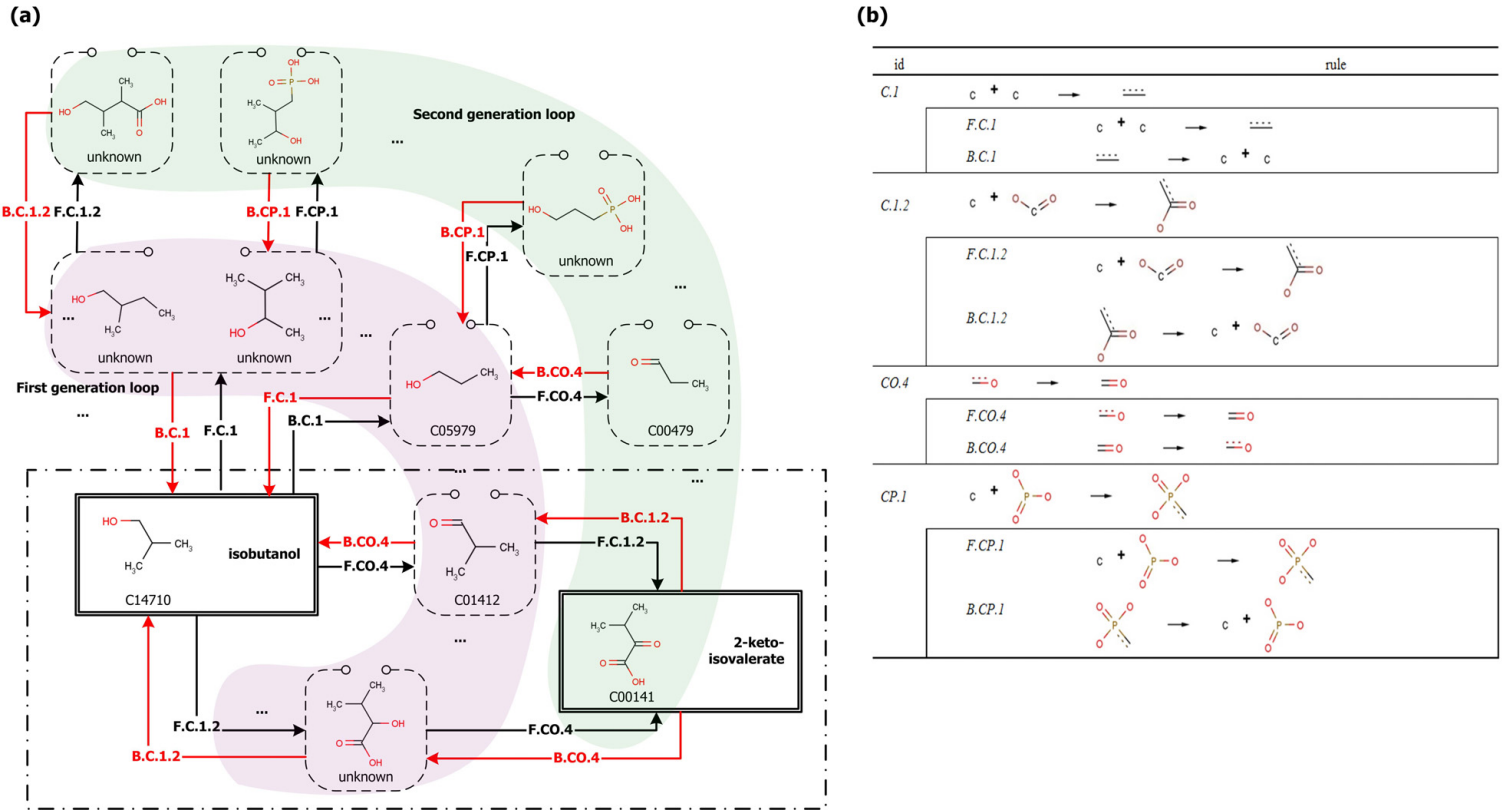
**Retrosynthesis example and the reaction rules**. (a) Part of the retrosynthetic tracing steps is shown while tracing loop limit is 2. If a substrate is produced from a target, the reverse of applied reaction rule is enrolled with the substrate and target. For the first generation inner loop, 62 primary substrate candidates are identified; in addition, 4,917 secondary substrate candidates are identified for the second generation outer loop. Among the substrate candidates, 233 compounds are identified in the KEGG database. 2-ketoisovalerate is selected as a starting chemical and the two viable routes are shown in the dashed box at the bottom. The compound ids are from KEGG database. (b) Reaction rules presented in (a) are listed. Every rule is basically defined as forward direction. For example, C.1 is exactly the same as F.C.1 while B.C.1 is defined as the reverse reaction of F.C.1.

**Figure 3:**
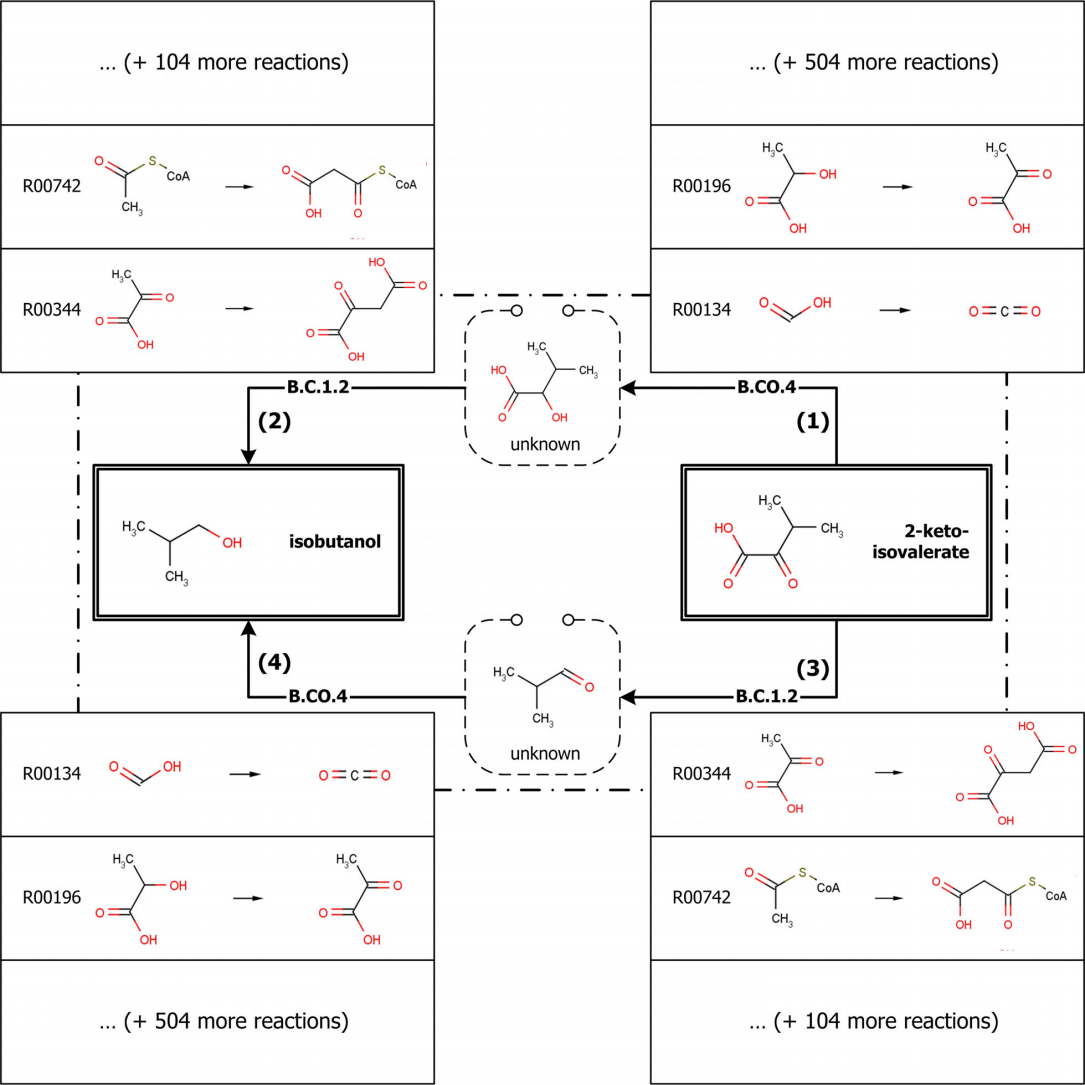
**The base routes and reaction route candidates for synthesis of isobutanol from 2-ketoisovalerate**. The reaction ids are from KEGG database.

Initially, for example, the structure of the target compound, isobutanol, was entered in the form of SMILES [17,18]. Then, predefined reaction rules were applied to the target compound for generating substrates. The generated substrates were regarded as intermediate target compounds, and the reaction rules were applied again. The recursive generation step was completed when the generation loop has reached the predefined limit. Throughout the loop, various routes for the production of target compound were generated. The system then detects known chemicals and displays them so as to select a starting compound (Figure 2a). The referred reaction rules are shown in Figure 2b. Among the detected known chemicals, 2-ketoisovalerate was selected as the starting compound. After the starting compound was determined, the routes from the starting compound to the target compound were retrieved for further analysis. The routes for the production of the target compound are defined as the base routes. The identified base routes for the synthesis of the isobutanol are shown in the bottom of Figure 2a. Using the mechanisms defined in every reaction step of base routes, the biochemical reactions in the KEGG database [19,20] were classified into the individual groups. Finally, all the routes from the starting compound to the target compound defined as the reaction route candidates were determined by combination of the reactions in each group. As shown in Figure 3, two base routes for the synthesis of isobutanol were identified and two groups, 506 reactions in B.CO.4 and 106 reactions in B.C.1.2, were classified for each base route. Finally, 107,272 reaction route candidates were determined.

**Figure 4:**
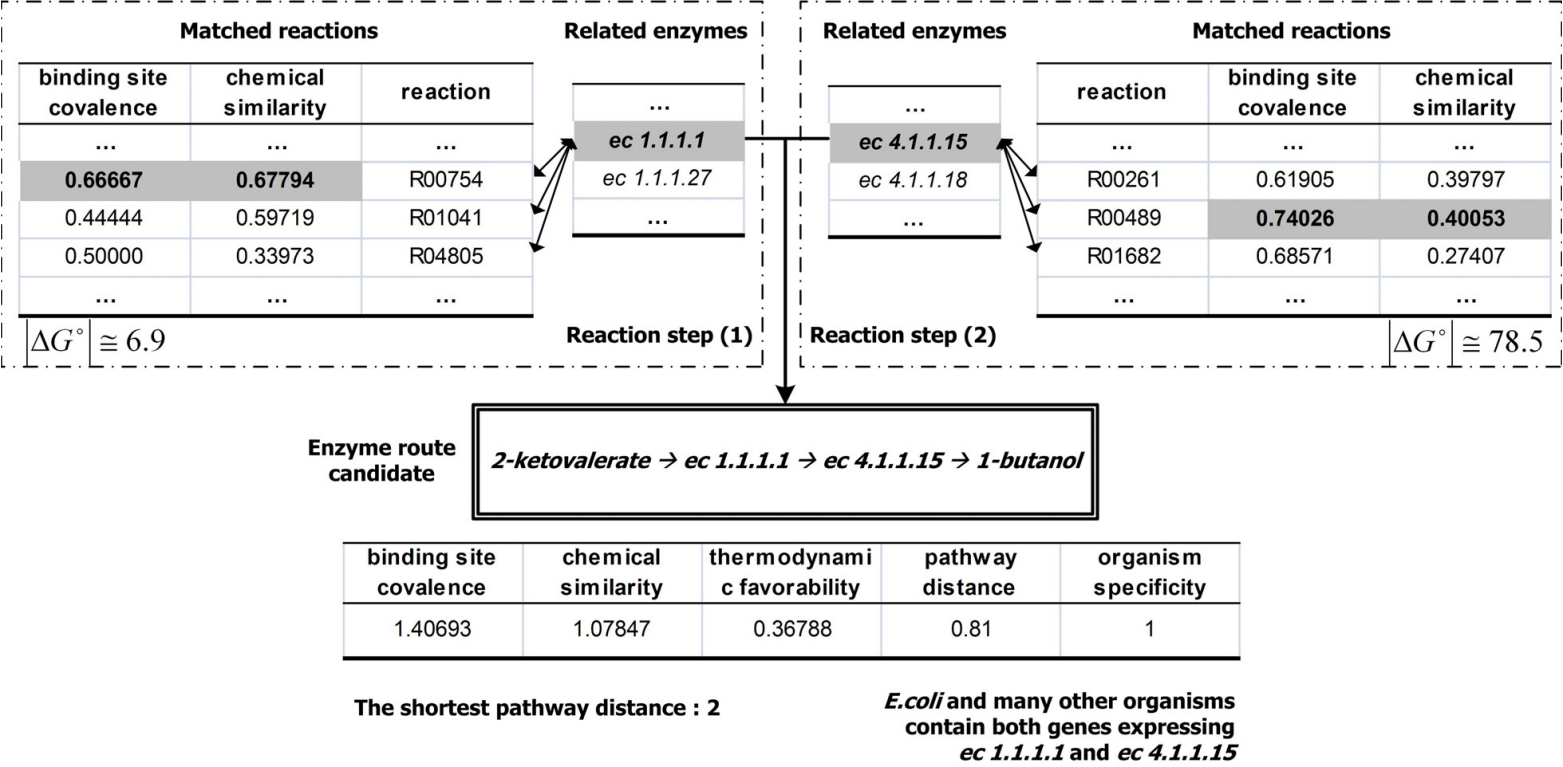
**Prioritization procedure of an enzyme route candidate**. One enzyme can be related with multi-reactions, for example, ec1.1.1.1 and ec4.1.1.15 catalyze three reactions, respectively. The best values of binding site covalence and chemical similarity of novel reaction steps are selected and evaluated to obtain those of enzyme route candidates.

Next, a prioritization method was applied to decide which route candidates are preferred by estimating five priority factors: binding site covalence, chemical similarity, thermodynamic favorability, pathway distance, and organism specificity. Throughout the prioritization step, reaction route candidates were rearranged into enzyme-based routes which were defined as enzyme route candidates. If multiple reactions are related to one enzyme, the values of binding site covalence and chemical similarity of the enzyme are determined by the highest values of those of the related reactions. Figure 4 shows the results of prioritization by the estimation of five factors for the synthesis of isobutanol.

Finally, the preference of each enzyme route candidate was determined as a score calculated by the weighted sum of each factor (Equation 6a in Methods). In the initial predictions, the six pathways were positioned within top 0.55% of the predicted candidates. Specially, isobutanol was within top 0.047% as the best result. From the initial prediction results, each three factor including thermodynamic favorability, pathway distance, and organism specificity is consistent for the six pathways. The other hand, the other factors including binding site covalence and chemical similarity are varied. It is caused that the same enzymes are applied to design the six pathways. Among the results of Atsumi *et al*. [16], the production rate of isobutanol was at least three times higher than the production rates of other higher alcohols. Also, the predicted rank for the synthetic pathway via KDC and ADH for the production of isobutanol is higher than other pathways. Therefore, the pathway was considered to be more suitable to produce isobutanol. For the accurate estimate of the relative influence of each factor which is expressed as a parameter, novel synthetic pathway for the production of isobutanol is selected.

**Figure 5:**
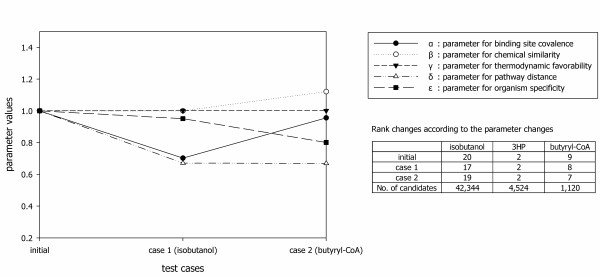
**Parameter and rank changes through evolutionary optimization**. The parameter changing behaviours are shown. According to the parameter changes, the rank changes of experimentally proven synthetic pathways for the production of isobutanol, 3HP, and butyryl-CoA are also presented.

To identify the optimal parameters, the evolutionary parameter optimization shown as Equation 7 in Methods was performed [21,22]. When the parameter values of 1 were used without optimization, the experimentally proven synthetic pathways of isobutanol was ranked at the 20th out of 42,344 enzyme route candidates. After the evolutionary parameter optimization, the parameter set was changed from {1, 1, 1, 1, 1} to {0.703, 1.000, 1.001, 0.671, 0.951} and the rank of the synthetic pathway was improved to the 17th (Figure 5). The ranks of six pathways were improved within top 0.42% of the predicted candidates than the initial predictions positioned within top 0.55% as mentioned above. In addition, there were enzyme route candidates that always have higher values in the final priority scores than those of the experimentally proven pathways (Table 1). It means that they have the superior values in all factors. Thus, the superior enzyme route candidates might be used as superior synthetic pathways for more efficient production of the target chemical.

**Table 1:** The superior enzyme route candidates for the production of each target chemical.

Target chemical	Enzyme route candidates
1-propanol	ec4.1.1.1 → ec1.1.1.2
	ec4.1.1.1 → ec1.1.99.8

isobutanol	ec4.1.1.1 → ec1.1.1.2
	ec4.1.1.1 → ec1.1.99.8

1-butanol	ec4.1.1.1 → ec1.1.1.2
	ec4.1.1.1 → ec1.1.1.61
	ec4.1.1.1 → ec1.1.99.8

2-methyl-1-butanol	ec 4.1.1.1 → ec1.1.1.2
	ec4.1.1.1 → ec1.1.99.8

3-methyl-1-butanol	ec 4.1.1.1 → ec1.1.1.2
	ec4.1.1.1 → ec1.1.99.8

2-phenylethanol	ec4.1.1.17 → ec1.1.1.90

3HP	ec4.2.1.17 → ec3.1.2.4

butyryl-CoA	ec1.2.7.7 → ec5.4.99.13

Enzyme route candidates have higher values in all factors compared to the experimentally identified pathways.

### Prediction of synthetic pathways for the production of 3HP

**Figure 6:**
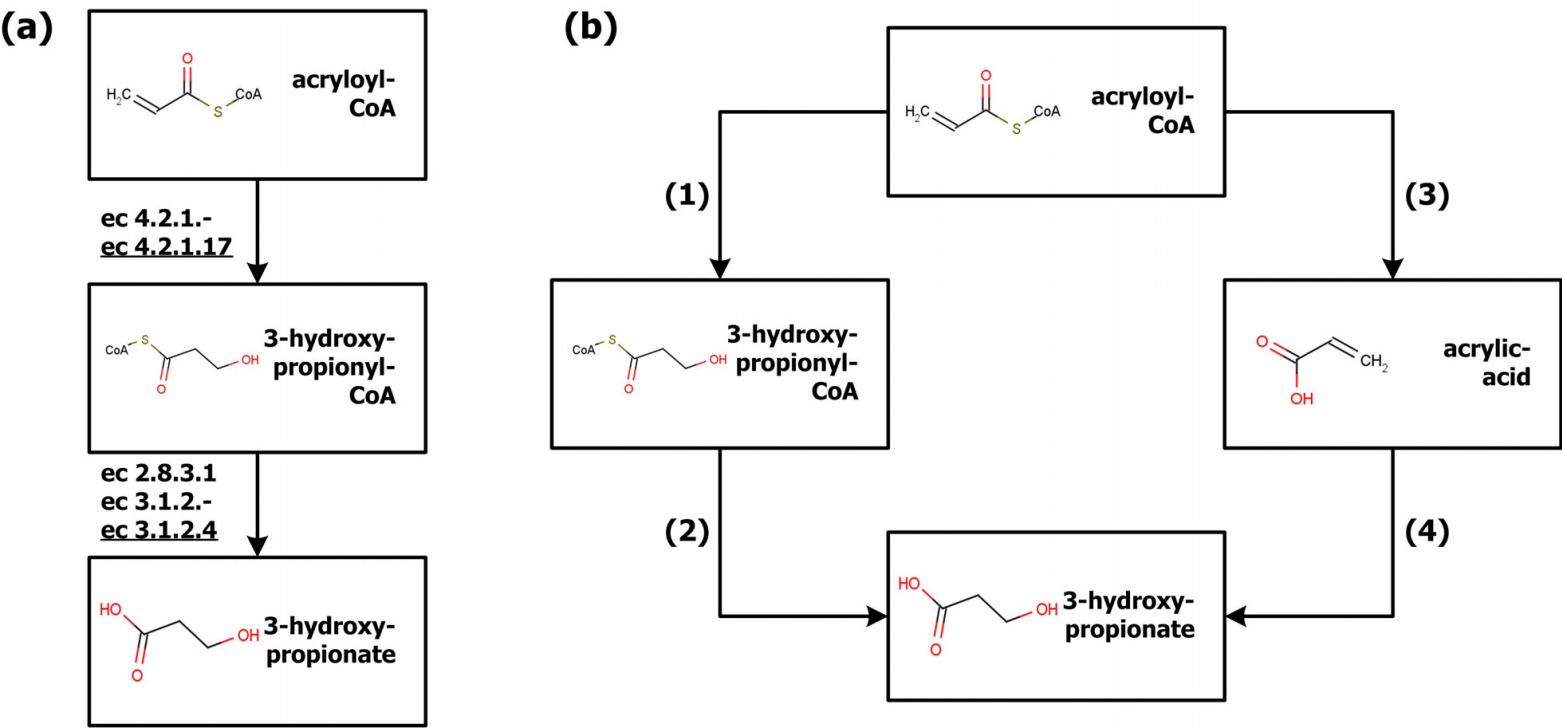
**3-hydroxypropionate synthetic pathways from acryloyl-CoA and involved enzymes**. (a) Experimentally validated synthetic pathways from acryloyl-CoA to 3-hydroxypropionate (3HP) where the enzymes existing in the KEGG database are underlined. (b) The base routes from acryloyl-CoA to 3HP. Pathway via acrylic-acid is identified additionally.

To examine the performance of the system framework developed in this study, the synthetic pathways for the production of 3HP were predicted as another example. 3HP is one of the top value added chemicals suggested by the U.S. Department of Energy [23]. Other than the suggested seven metabolic pathways for the production of 3HP from glucose [24,25], alternative pathways from acryloyl-CoA were identified. The structure of 3HP and its existing and alternative synthetic pathways from acryloyl-CoA are shown in Figure 6a. To identify the synthetic pathways for the production of 3HP from acryloyl-CoA, two base routes shown in Figure 6b and 29,592 reaction route candidates were generated. Next, reaction route candidates were further analyzed quantitatively in order to identify more promising routes by employing a prioritization algorithm. During the prioritization, 4,524 enzyme route candidates were generated and evaluated. The experimentally proven synthetic pathway from acryloyl-CoA to 3HP through ec4.2.1.17 and ec2.8.3.1 was ranked at the second out of 4,524 enzyme route candidates (within top 0.044%). Since the existing pathway is superior enzyme route candidate of the experimentally proven pathway, evolutionary optimization does not change the parameter values and the rank. This result demonstrates the efficiency and reliability of the developed system framework for identifying novel synthetic pathways for the production of desired chemicals.

### Prediction of alternative synthetic pathways for the production of 1-butanol

**Figure 7:**
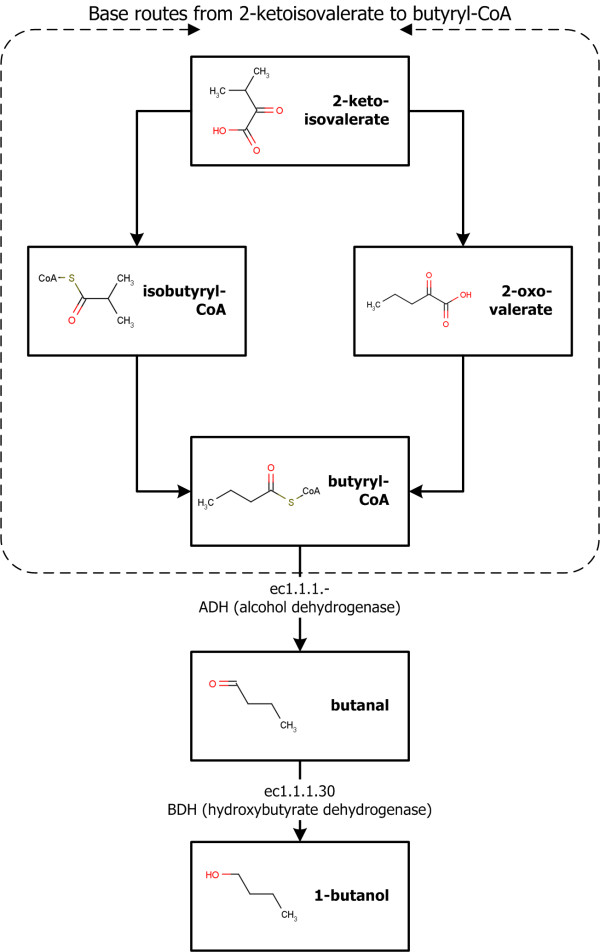
**Alternative synthetic pathways for the production of 1-butanol via butyryl-CoA**. The base routes to design novel synthetic pathway are blocked by dashed box while the existing pathway from butyryl-CoA to 1-butanol is shown below.

In this paper, the alternative synthetic pathway for the production of 1-butanol from 2-ketovalerate [16], as Atsumi *et al*. presented, has been identified. Since the two step synthetic pathway for the production of 1-butanol from butyryl-CoA already exists in KEGG database, the synthetic pathways to the butyryl-CoA were explored. The constructed alternative pathways with base routes are shown in Figure 7. During the route generation process, 3,240 reaction route candidates and 1,120 enzyme route candidates were identified. The enzyme route candidates are quantitatively analyzed by the prioritization method and the resulting top 10 pathways are shown in Table 2. Among the top 10 pathways, the 8th ranked enzyme route candidate, a novel pathway *via *ec1.2.1.25 and ec5.4.99.13, was successfully synthesized to produce butyryl-CoA from 2-ketoisovalerate. This pathway is structurally identical compared to the existing pathway; however, no organism has been found to produce the butyryl-CoA *via *the enzymes. In other words, the pathway obtained lower score in the organism specificity. That is the reason why the pathway is ranked at the 8th. After the evolutionary optimization, the rank is slightly changed to the 7th and the parameter set is changed to {0.956, 1.121, 1.001, 0.669, 0.801}. Through this work, the novel alternative synthetic pathway for the production of 1-butanol has been identified. This study demonstrates the applicability of the developed method and system framework.

**Table 2:** Top 10 enzyme route candidates for the synthesis of butyryl-CoA and the prioritization values.

Rank	Enzyme route candidates	Intermediate	***XB***	***XC***	***XT***	***XP***	***XO***	***X***
1	ec1.8.1.4 → ec5.4.99.13	isobutyryl-CoA	2.000	1.855	0.449	0.900	1.000	2.633

1	ec2.3.1.12 → ec5.4.99.13	isobutyryl-CoA	2.000	1.855	0.449	0.900	1.000	2.633

1	ec1.2.4.1 → ec5.4.99.13	isobutyryl-CoA	2.000	1.855	0.449	0.900	1.000	2.633

4	ec1.2.4.2 → ec5.4.99.13	isobutyryl-CoA	2.000	1.849	0.449	0.810	1.000	2.600

5	ec1.2.7.1 → ec5.4.99.13	isobutyryl-CoA	2.000	1.950	0.449	0.900	0.531	2.457

6	ec1.2.7.7 → ec5.4.99.13	isobutyryl-CoA	2.000	2.000	0.449	0.900	0.478	2.457

7	ec2.3.1.54 → ec5.4.99.13	isobutyryl-CoA	2.000	1.950	0.449	0.810	0.531	2.427

8	ec1.2.1.25 → ec5.4.99.13	isobutyryl-CoA	2.000	2.000	0.449	0.900	0.387	2.414

9	ec5.3.1.17 → ec1.2.4.2	2-oxo-valerate	1.900	1.605	0.368	0.810	1.000	2.402

10	ec5.3.1.17 → ec1.2.7.3	2-oxo-valerate	1.900	1.588	0.368	0.810	1.000	2.393

XB is the binding site covalence, XC is the chemical similarity, XT is the thermodynamic favorability, XP is the pathway distance, and XO is the organism specificity. The final priority X is evaluated by Equation 6a with the previously estimated parameter set {0.703, 1.000, 1.001, 0.671, 0.951}. 6th ranked enzyme route candidate is the superior set of the experimentally proven pathway which is ranked at the 8th.

## Conclusions

In this study, a system framework was established to identify promising enzyme candidates to synthesize desired chemicals. This approach can also be applied to find the novel pathways for the biodegradation of chemicals. Through this work, 50 reaction rules representing numerous biochemical reactions were set up for qualitative analysis. The most notable feature of the study is the development of a new quantitative analysis method, prioritization scoring algorithm. Using the novel estimation methods, new opportunities of enzymes can be predicted with greater precision. Moreover, the parameters are estimated by an evolutionary optimization method, and thus more accurate scores can be estimated as more experimentally validated data are added. This *in silico *prediction system is expected to contribute significantly to *in vivo *or *in vitro *experiments.

## Methods

### System framework

**Figure 8:**
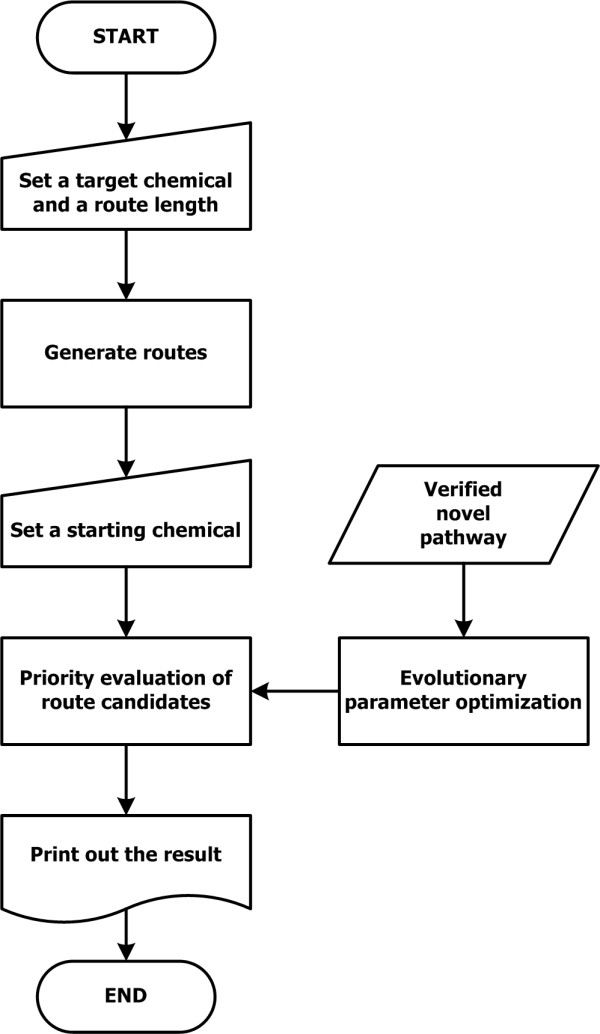
**The system flow diagram**. The system flow is shown in the dashed box while the research cycle is connected with the steps in system flow. The research cycle explains how the system is developed and updated.

To identify promising enzyme candidates that catalyze novel biochemical reactions to synthesize desired chemicals, a system framework that follows the steps of target compound definition, route generation, prioritization, and parameter optimization was developed (Figure 8). The detailed procedure is shown in the Results and Discussion using the isobutanol synthesis pathways as examples. Having set isobutanol as a target chemical, the route length was appointed as 2. As shown in Figure 2, the predefined 50 reaction rules were applied twice repeatedly since the route length was 2; the reaction rules are explained in Database construction. Then, the substrate candidates were detected and a starting chemical has been selected from the candidates. Following this step, enzymatic reactions having the same reaction rule with the reactions in the pathway from the starting chemical to the target chemical were matched. Then the prioritization method based on quantitative analysis has been performed to identify which enzymes would be more promising for the desired novel pathway; the prioritization method is explained in Prioritization. As a result, the combinations of enzymes were sorted in decreasing order of the prioritization scores. If a novel pathway is proven by further studies including experimental validation, the evolutionary parameter optimization is performed to update the parameters based on the experimental data; the parameter optimization is explained in Parameter optimization.

JChem [26] was imported to handle chemical structures and GAMS/CPLEX [27,28] were used to perform parameter optimization. KEGG was employed as a pathway reference database [19,20]. SMILES/SMARTS [17,18] were used as chemical structure representation languages, JAVA as a programming language, and MSSQL 2005 Server were used as a database server.

### Database construction

**Figure 9:**
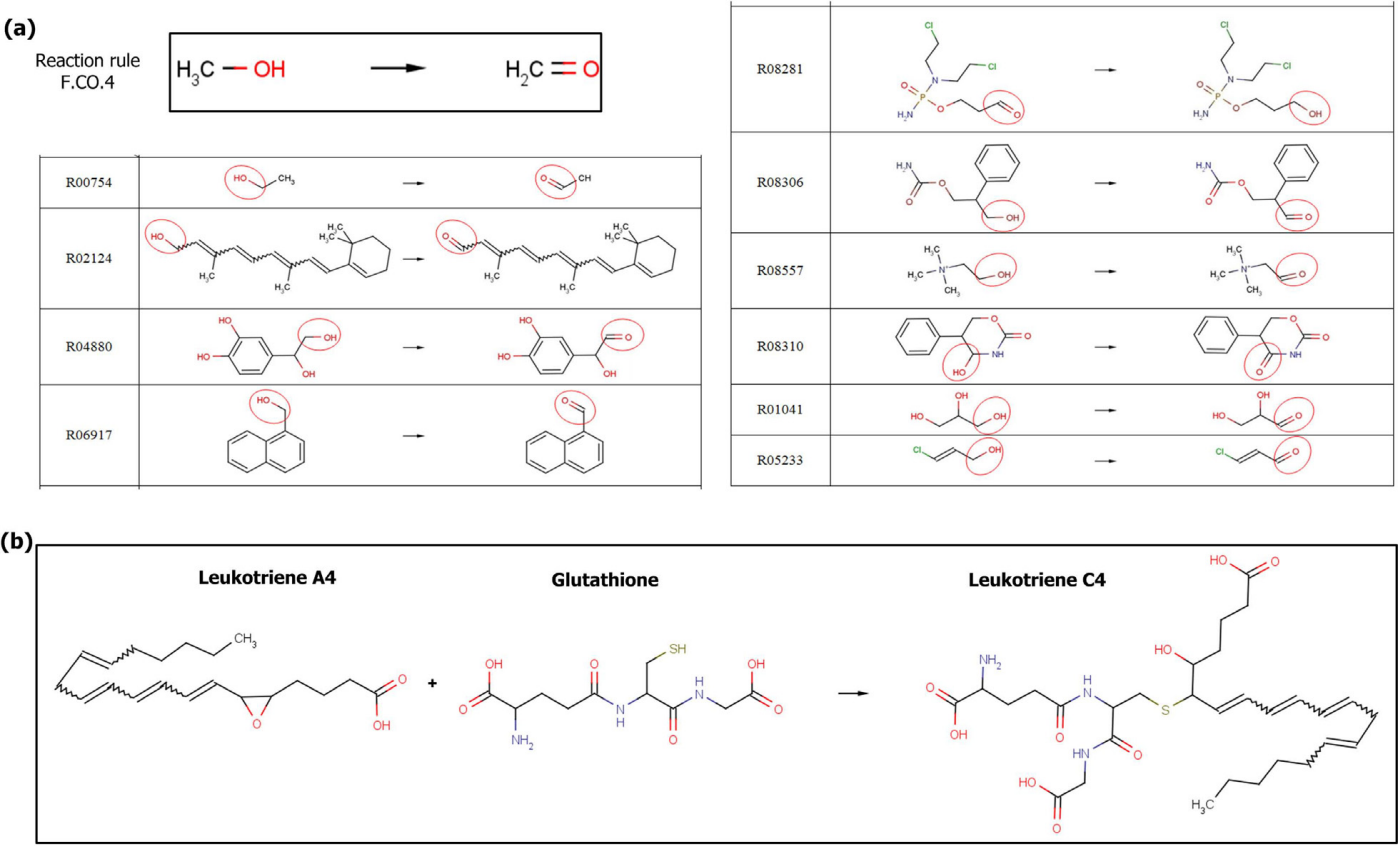
**Examples of a reaction rule with related reactions and a complicated reaction**. (a) Main substrate and product of reactions catalyzed by ADH (EC1.1.1.1) are listed. The reaction rule explains every reaction in both of forward and backward directions. (b) An example of complicated reaction (leukotriene-A4-forming reaction) which is catalyzed by ec4.4.1.20. The reaction mechanism is hard to be defined as a rule.

**Figure 10:**
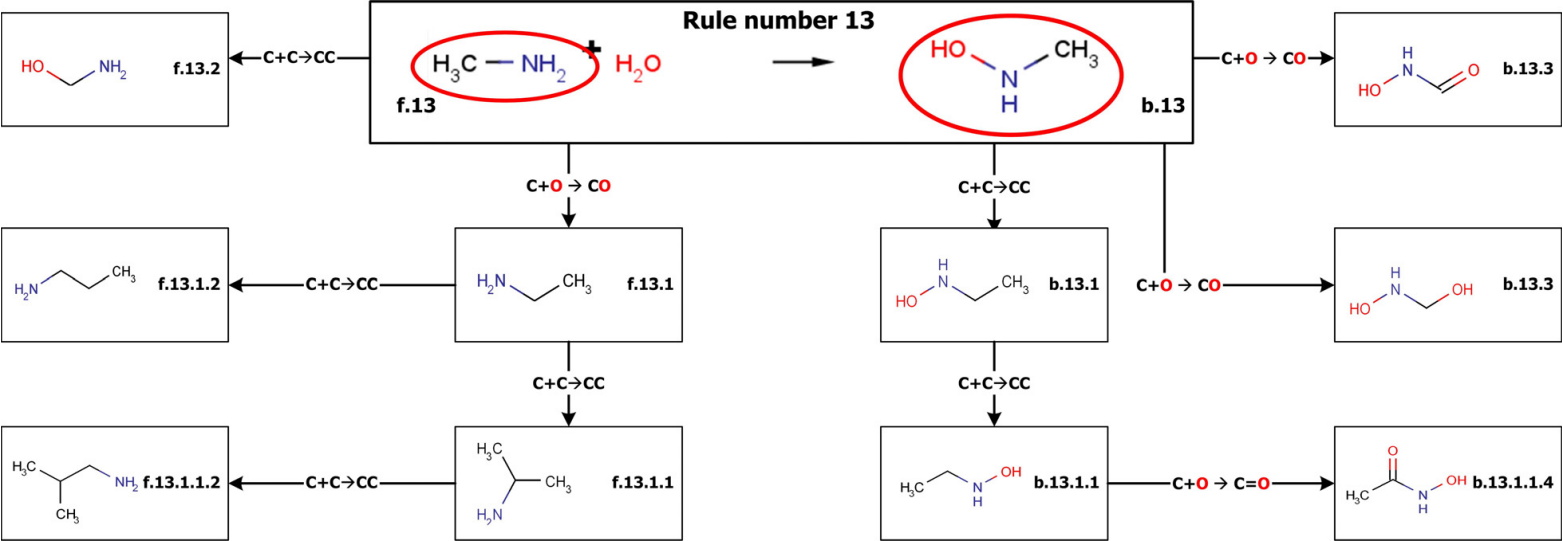
**An example of a binding site rule and naming**. Based on the oxygen attaching reaction rule (rule number: 13), the functional groups on the both sides of the reaction is extended. The systematic names are also extended based on the direction and the rule id.

For the management of data for pathway/enzyme prediction, two databases were developed: reaction rule database and binding site rule database. First, the reaction rules explaining reaction mechanisms were defined. Basically, a reaction rule is constructed based on the smallest substructure related to the structural change of the main reactant and product by the reaction. Thus, cosubstrates or cofactors are not considered. With the small number of reaction rules, various reactions can be described. In this study, 50 reaction rules were constructed (See additional file 1: The list of 50 reaction rules). As shown in Figure 9a, several reactions catalyzed by ADH (alcohol dehydrogenase; ec1.1.1.1) can be explained by a single reaction rule defined as F.CO.4. After the construction of reaction rules, all the reactions in the database were classified according to the reaction rules. The reaction rules are applied not only to the forward direction, but also to the backward direction. In addition, reaction rules can be combined in order to represent one reaction. As a result, these simple reaction rules can represent 81% of enzymes present in the KEGG database. Reactions are not categorized when the detailed structure of compounds in reactions does not defined-; when compounds of reactions are not carbon-related- or polymer-related; or when the reaction mechanism cannot be defined clearly in terms of the structure changes of both directions, especially some lyases and ligases cannot be identified clearly. Figure 9b shows the example of leukotriene-A4-forming reaction catalyzed by ec4.4.1.20. The backward reaction was defined by cleaving the C-S bond, by the forward direction could not be defined clearly with simple and widely used cosubstrates such as carbon dioxide. The database for binding site rules has been constructed to estimate binding site covalence among the prioritization factors. Since a binding site is defined based on the three dimensional structure of a molecule, it is difficult to identify a binding site with its structural formula alone, without stereo information [29]. To deal with this problem, similar chemical substructures including the functional group may form similar binding sites [29-31]. For each reaction rule, the branch was extended or a ring was generated based on the functional group to generate chemical substructures which are defined as the binding site rules in this paper. The branch was extended with 103 atom attaching reactions or a ring is generated with 25 ring generation reactions when the main branch of the chemical had 3 or more atoms. Next, binding sites rules are named with the direction and the related reaction rule id for further estimation of binding site covalence. For the binding site rule on the substrate side of a reaction rule, the initial step is 'f'. Otherwise, the initial step is 'b' for the binding site on the product side. The letters 'f' and 'b' denote 'forward' and 'backward' direction of the reaction rule, respectively. Then, an index of reaction rule represented by a number is added to distinguish where the binding site rule is defined. Each step is divided by a dot. Figure 10 presents an example. As the binding site rule grows, the name is extended. The remained number represents the hierarchical information of the binding site rule.

### Prioritization

If an enzyme reaction has the same reaction rule with a novel reaction in a desired pathway, then it is matched as a similar reaction. There can exist many matched enzymatic reactions for each reaction in the desired pathway, and thus it is necessary to clarify which enzymatic reactions will be more promising. To address this problem, the similar reactions need to be evaluated quantitatively. The quantitative aspect of likeness is defined by a scoring algorithm - referred to as prioritization. The prioritization method is composed of five factors: binding site covalence, chemical similarity, thermodynamic favorability, pathway distance and organism specificity. Binding site covalence and chemical similarity are evaluated by comparing two reactions. Here, the binding site rules were defined by extending functional groups that are occupied in every reaction rule. The binding site covalence represents the local similarity between two molecules whereas the chemical similarity is estimated based on the entire structures of molecules in each reaction route candidate. Thermodynamic favorability is estimated by chemical structure changes through identified base routes. Pathway distance and organism specificity measure the relationships among enzymes to catalyze reactions in each enzymatic synthetic route from starting to target chemical. Those factors are used to calculate priorities of the enzyme route candidates.

#### Binding site covalence

Binding site covalence describes how similar two reactions are from the point of chemical structure changes. As described above, binding site rules had been defined as substructures of chemicals including their functional groups. In addition, the systematic name of a binding site rule entails the trace of branch extension. Therefore, the binding site covalence is calculated by systematic names; the more similar names the binding site rules have, the more similar structures they have. The binding site covalence between a known reaction in a reaction route candidate and a novel reaction in a base route can be estimated as follows. First, the covalences of molecules on the substrate side and the product side are evaluated. The binding site covalence is then calculated by summation of the covalence of molecules on both sides of reactions.(1a)

where

*i*   : index of reaction steps in a base route, (*i *= 1,2,...,*n*)

*n*   : number of reaction steps in a base route (route length)

*j*, *J*   : index and set of enzyme route candidates, respectively (∀*j *∈ *J*)

*XB*, *XB_j_*, *XB_i_*   : binding site covalence of an enzyme route candidate, a reaction route candidate and a reaction step in the base route, respectively

: system name steps in common for two reactions on the substrate side and the product side, respectively

: system name steps of a novel reaction *f*_*i *_on the substrate side and the product side for a reaction step *i*, respectively

: system name steps of a known reaction *g*_*i *_on the substrate side and the product side for a reaction step *i*, respectively

**Figure 11:**
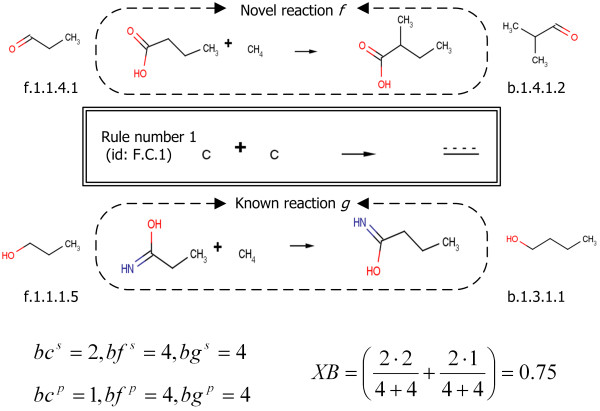
**An example of estimation of binding site covalence**. The binding site covalence between novel and known reactions belonging to rule number 1 (rule id F.C.1) is evaluated. The largest binding site, a compound contains, is shown near by the compound. Hereby, *b*_*c *_means system name step in common for reaction *f *and *g *while *b*_*f *_and *b*_*g *_means system name steps of a novel reaction *f *and a known reaction *g*, respectively. The superscript *s *and *p *means substrate side and product side, respectively. *XB *is the binding site covalence between a novel reaction *f *and a known reaction *g*. (Equation 1a).

An example is shown in Figure 11. One base route can be compared to several reaction route candidates so as to identify which reaction route candidate will be more similar in terms of local structures around functional groups. If chemicals have the same binding site rule as the largest substructure on both sides of the reactions, the binding site covalence of the reaction step becomes the maximum value, one, while the binding site covalence is normalized between zero and one.

#### Chemical similarity

**Figure 12:**
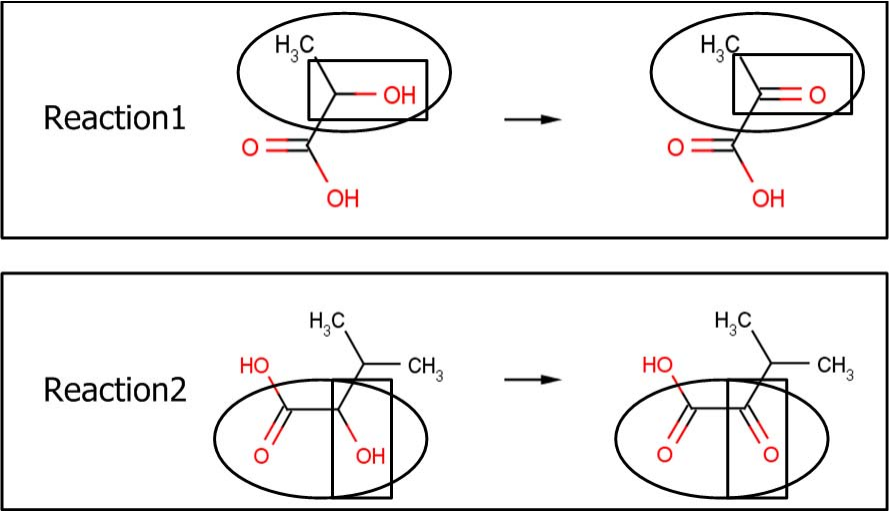
**An example of main chemical changes in two reactions**. Structures in squares indicate functional groups and structures in circles indicate binding sites for each chemical.

Chemical similarity is examined on both sides of reactions with respect to how similar two reactions are in terms of chemical fragments [32]. The chemical similarity represents the similarities of the entire molecular structures, while the binding site covalence identifies only the substructure similarities. In Figure 12, the binding site covalence is calculated by considering the circled structures only; on the other hand, the chemical similarity is calculated by considering the whole structures. The binding site shown in the circle always contains a functional group shown in the square. In this case, the binding site covalence might get higher value than the chemical similarity since the substructure similarities on both sides of reactions are more significant. Tanimoto coefficient, representing chemical dissimilarity based on a fragment analysis using chemical bit-strings was employed [32,33]. The Tanimoto coefficient has been one of the most popular methods to show chemical dissimilarity, since it evaluates the relationship between chemicals accurately, is normalized from zero to unity and easy to use. In the system framework, the complementary value of the Tanimoto coefficient was used to evaluate the similarity not the dissimilarity. In the same manner as the binding site covalence calculation, the values are estimated on both sides of the reactions and the summation is taken for each reaction step.(2a)

where

*XC*, *XC_j_*, *XC*_*i *_: chemical similarity of an enzyme route candidate, a reaction route candidate and a reaction step in the base route, respectively

*T*(*f*, *g*): Tanimoto coefficient for two molecules and while 0 ≤ *T*(*f*, *g*) ≤ 1

: substrate and product of a reaction step in a base route *f*_*i *_for a step *i*, respectively

: substrate and product of a reaction step in a reaction route candidate *g*_*i *_for a step *i*, respectively

#### Thermodynamic favorability

**Figure 13:**
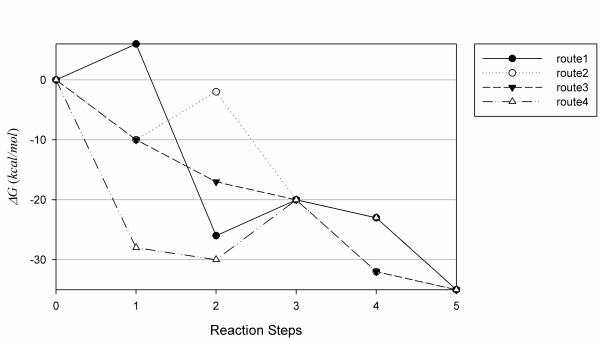
**Gibbs free energy of formation changes via five routes**. Route 3 is preferred to others since it has less fluctuation.

The group contribution method was used to estimate Gibbs free energy of formation [34,35]. Normally, Gibbs free energy of formation has been used to determine whether a reaction is thermodynamically feasible, and it takes a negative value if the reaction is feasible. Since the group contribution method requires only group and atom properties of a molecule, it is appropriate to be adopted in the system framework. However, the environmental parameters in a living system, such as temperature and pressure, are not ideal and change continuously, and thus the group contribution method cannot estimate the exact Gibbs free energy of formation. Therefore, we employed the Gibbs free energy of formation as a reference to determine thermodynamic favorability. From the starting to target chemical, the overall Gibbs free energy of formation estimated by the group contribution method must be the same. Thus, the more important variable than the overall Gibbs free energy of formation is the energy level fluctuation through the synthetic route (Figure 13). As shown in the Equation 3a, the absolute values of Gibbs free energies of formations are summed through a base route so as to obtain the 1-norm distance [36].(3a)

where

*XT*   : normalized thermodynamic favorability of an enzyme route candidate

*XT*_*i*_   : Gibbs free energy of formation of a step in a base route

*XT*_*j*_   : thermodynamic favorability of an enzyme route candidate

Here, we adjusted the range of thermodynamic favorability from zero to one for comparison with other factors. Moreover, a larger value indicates more fluctuation so that the values should be converted. To make a larger value more favorable, the negative exponential function of each thermodynamic favorability ratio with respect to the maximum value was applied. After the three factors have been estimated, all the identified routes are rearranged as enzyme route candidates. If two or more reactions are catalyzed by one enzyme, then the best values of binding site covalence and chemical similarity are selected (Equation 1c, 2c).

#### Pathway distance

Previous studies have been attempted to identify the genomic relations of pathway distance [37,38]. The pathway distance was initially defined as the shortest path between two enzymes; if two enzymes catalyze same reaction, then the pathway distance becomes zero. Shorter pathway distance showed higher genomic relation based on gene pairs [38]. The shortest pathway distance between two enzymes is examined by a full search starting from one enzyme until met the other one. First, the pathway distance between two enzymes is evaluated and then that of the second and third enzymes are evaluated again until the last enzyme in the enzyme route candidate is encountered.(4a)

where

*XP*   : pathway distance of an enzyme route candidate

*XP*_*i*,*i*+1_   : revised pathway distance between *i *th and (*i *+ 1) th steps in an enzyme route candidate

*p*_*i*,*i*+1 _   : pathway distance between *i *th and (*i *+ 1) th steps in an enzyme route candidate

Finally, all the evaluated pathway distances between steps in an enzyme route candidate are multiplied. If one distance is increased, the co-expression probability of two enzymes is decreased (Equation 4a).

#### Organism specificity

**Figure 14:**
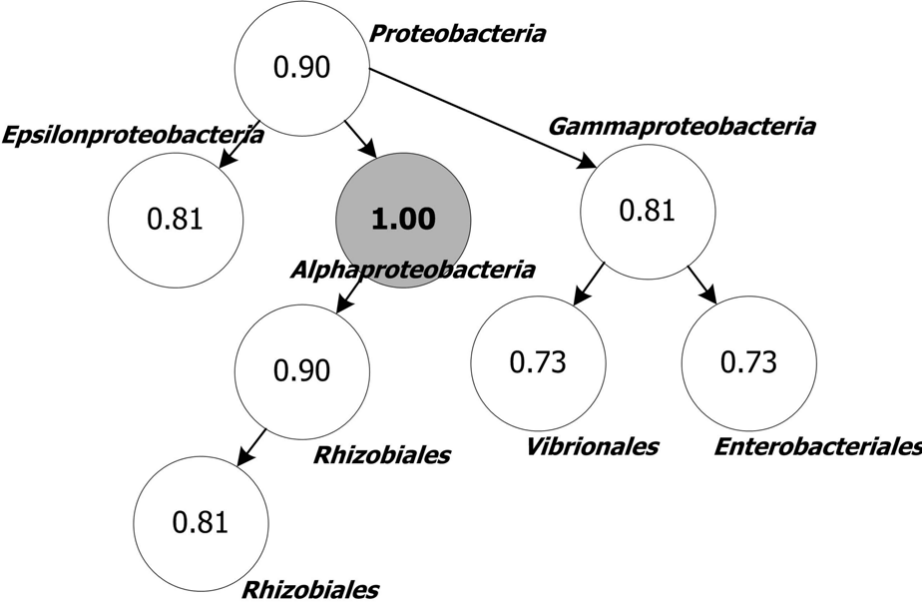
**Estimated organism specificities in a hierarchical tree**. This tree represents lineage relationships among organisms. The value written in each node indicates the organism specificity compared to the filled node. If an enzyme a is expressed in *Alphaproteobacteria *and an enzyme b is expressed in *Enterobacteriales*, the organism specificity between a and b is 0.73.

Organism specificity has been assigned in the same manner as pathway distance estimation. It estimates the organism-to-organism distance, a subject that has been studied extensively [39-41]. The distance is calculated based on the hierarchical information of organism lineages based on gene changes from generation to generation. If an enzyme is one generation differs from the other one, the co-expression probability of the two enzymes is decreased (Figure 14, Equation 5a). The lineage information of all genes stored in the KEGG database was used. The distance is established by comparing the lineage information; moreover, the system explores the closest organisms which encode the two enzymes.(5a)

where

*XO*   : organism specificity of an enzyme route candidate

*XO*_*i*,*i*+1 _   : organism specificity between *i*th and (*i *+ 1) th step in an enzyme route candidate

*o*_*i*_   : number of lineage generations of *i*th step in an enzyme route candidate

*o*_*i*,*i*+1 _   : number of lineage generations in common for *i*th and (*i *+ 1) th step in an enzyme route candidate

#### Final priority score

The meanings of five factors addressed above are categorized into three groups: structural similarity of reaction steps in a route, thermodynamic benefits among intermediates and co-expression probability of enzymes. With those factors, the priority score for each route is finally obtained by Equation 6a.(6a)

where

*X*: priority score of an enzyme route candidate

*α*   : parameter for binding site covalence

*β*   : parameter for chemical similarity

*γ*   : parameter for thermodynamic favorability

*δ*   : parameter for pathway distance

*ε*   : parameter for organism specificity

Each parameter was initially set to be one; in addition, the parameters were optimized, as introduced in the following section. Finally, the promising enzyme candidates are sorted by the priorities where a higher priority value means greater likelihood to catalyze a novel synthetic route. Since numerous enzyme candidates are ordered quantitatively, promising enzyme candidates among them could be distinguished and applied to experiments.

#### Parameter optimization

Finding optimal parameters to identify the best candidate is one of the critical issues in determining valid priority, since the parameters denote the impacts and the relationships of the aforementioned five factors. To optimize the parameters, the known enzymes, exist in the KEGG database, were utilized to identify the experimentally proven novel pathways. With pathway information in the KEGG database, the currently known pathways will inevitably receive the highest scores. For this reason, the criteria were designed based on currently known pathways. Herein, the compared chemical structures between a synthesized route and an existing pathway are identical, and thus the binding site covalence and chemical similarity must be the maximum values for each step in a route. Moreover, pathway distance must be short and the organism specificity is one. Thermodynamic favorability can be better for an unknown route; however, this effect cannot be analyzed alone without considering other factors. To validate our approach, the parameters have been optimized by satisfying the criterion that newly synthesized pathways from the literatures are to be ranked high. Using the data set of prioritization factors, a mixed integer linear problem was set up as follows. The primary objective is to minimize the difference between the priority score of a desired route candidate with current parameters and the score with adjusted parameters. The secondary objective is to maximize the number of reactions having lower scores than the desired route candidate. That is, the parameters evolve whenever a test case is added so as to adjust parameters that minimize the deviation and make experimentally verified pathways be ranked high simultaneously.(7a)

where

*α'*   : adjusted parameter for binding site covalence

*β'*   : adjusted parameter for chemical similarity

*γ'*   : adjusted parameter for thermodynamic favorability

*δ'*   : adjusted parameter for pathway distance

*ε'*   : adjusted parameter for organism specificity

*XB*^*obj*^: binding site covalence of the desired candidate *obj*

*XC*^*obj*^   : chemical similarity of the desired candidate *obj*

*XT*^*obj*^   : thermodynamic favorability of the desired candidate *obj*

*XP*^*obj*^   : pathway distance of the desired candidate *obj*

*XO*^*obj*^   : organism specificity of the desired candidate *obj*

   : priority score of a route candidate *j *with adjusted parameters

   : priority score of the desired candidate *obj *with adjusted parameters

*y*_*j*_   : binary variable, 

The priority scores are calculated by Equation 6 while the lower bound and upper bound of parameters are 0.5 and 1.5, respectively. This problem was solved by the parameter evolutionary optimization [21,22]. After the evolutionary parameter estimations were performed with five test cases sequentially, the parameters have been adjusted, and consequently the Equation 6a was specified to Equation 6b.(6b)

The evolutionary parameter optimization process according to enrolled test cases is presented in Figure 5.

## Authors' contributions

SYL and SP designed and directed the research. AC designed the methodology and implemented the system framework. HY designed the methodology and supported the implementation. JHP performed experiments to construct the synthetic pathway for the production of 1-butanol from 2-keto-isovalerate via butyryl-CoA. All authors wrote the manuscript. All authors read and approved the final manuscript.

## Supplementary Material

Additional file 1 **The list of 50 reaction rules (reactionRules.xls)**. The alphabets of rule IDs show the atoms participating in the functional groups on the substrate side. The first number in the ID is assigned according to the rule shown in the next column. The reaction mechanisms and the structures of cosubstrates and coproducts are represented by SMARTS and SMILES, respectively. SMILES is the chemical structure representation language and SMARTS is its query language. If the reaction mechanisms of two reaction rules are the same and the cosubstrates are different, an extra number is shown. In the cosubstrate or the coproduct column, 'itself' means that two same chemicals participate in the reaction. Click here for file

## References

[B1] Arita M Metabolic reconstruction using shortest paths Simulat Pract Theory 2000 8 109 125 10.1016/S0928-4869(00)00006-9

[B2] Feldman HJ Dumontier M Ling S Haider N Hogue CW CO: A chemical ontology for identification of functional groups and semantic comparison of small molecules FEBS Lett 2005 579 4685 4691 10.1016/j.febslet.2005.07.039 16098521

[B3] McShan DC Rao S Shah I PathMiner: predicting metabolic pathways by heuristic search Bioinformatics 2003 19 1692 1698 10.1093/bioinformatics/btg217 12967966 PMC2709535

[B4] McShan DC Shah I Heuristic search for metabolic engineering: de novo synthesis of vanillin Comput Chem Eng 2005 29 499 507 10.1016/j.compchemeng.2004.08.038

[B5] Klopman G Dimayuga M Talafous J META. 1. A program for the evaluation of metabolic transformation of chemicals J Chem Inf Comput Sci 1994 34 1320 1325 7989397 10.1021/ci00022a014

[B6] Pharkya P Burgard AP Maranas CD OptStrain: a computational framework for redesign of microbial production systems Genome Res 2004 14 2367 2376 10.1101/gr.2872004 15520298 PMC525696

[B7] Darvas F Predicting metabolic pathways by logic programming J Mol Graph 1988 6 80 86 10.1016/0263-7855(88)85004-5

[B8] Greene N Judson PN Langowski JJ Marchant CA Knowledge-based expert systems for toxicity and metabolism prediction: DEREK, StAR and METEOR SAR QSAR Environ Res 1999 10 299 314 10.1080/10629369908039182 10491855

[B9] Hou BK Wackett LP Ellis LB Microbial pathway prediction: a functional group approach J Chem Inf Comput Sci 2003 43 1051 1057 12767164 10.1021/ci034018f

[B10] Karp PD Paley S Romero P The pathway tools software Bioinformatics 2002 18 225 232 10.1093/bioinformatics/18.suppl_1.s225 12169551

[B11] Hatzimanikatis V Li C Ionita JA Henry CS Jankowski MD Exploring the diversity of complex metabolic networks Bioinformatics 2005 21 1603 1609 10.1093/bioinformatics/bti213 15613400

[B12] Ihlenfeldt WD Gasteiger J Computer-assisted planning of organic syntheses: the second generation of programs Angew Chem Int Ed Engl 1996 34 2613 2633 10.1002/anie.199526131

[B13] Li C Henry CS Jankowski MD Ionita JA Hatzimanikatis V Broadbelt LJ Computational discovery of biochemical routes to specialty chemicals Chem Eng Sci 2004 59 5051 5060 10.1016/j.ces.2004.09.021

[B14] Prather KL Martin CH De novo biosynthetic pathways: rational design of microbial chemical factories Curr Opin Biotechnol 2009 19 468 474 10.1016/j.copbio.2008.07.009 18725289

[B15] Rodrigo G Carrera J Prather KL Jaramillo A DESHARKY: automatic design of metabolic pathways for optimal cell growth Bioinformatics 2008 24 2554 2556 10.1093/bioinformatics/btn471 18776195

[B16] Atsumi S Hanai T Liao JC Non-fermentative pathways for synthesis of branched chain higher alcohols as biofuels Nature 2008 451 86 89 10.1038/nature06450 18172501

[B17] Weininger D SMILES, a chemical language and information system. 1. Introduction to methodology and encoding rules J Chem Inf Comput Sci 1988 28 31 36

[B18] Weininger D Weininger A Weininger JL SMILES: 2. Algorithm for generation of unique SMILES notation J Chem Inf Comput Sci 1989 29 97 101

[B19] Kanehisa M Goto S KEGG: Kyoto Encyclopedia of Genes and Genomes Nucleic Acids Res 2000 28 27 30 10.1093/nar/28.1.27 10592173 PMC102409

[B20] Ogata H Goto S Sato K Fujibuchi W Bono H KEGG: Kyoto Encyclopedia of Genes and Genomes Nucleic Acids Res 1999 27 29 34 10.1093/nar/27.1.29 9847135 PMC148090

[B21] Sarker R Mohammadian M Yao X Evolutionary Optimization 2002 Springer

[B22] Segre D Vitkup D Analysis of optimality in natural and perturbed metabolic networks Proc Natl Acad Sci USA 2002 99 15112 15117 10.1073/pnas.232349399 12415116 PMC137552

[B23] Werpy T Petersen G Top value added chemicals from biomass Volume I - Results of screening for potential candidates from sugars and synthesis gas [Electronic Version] DOE Science and Technology Information 2004

[B24] Gokarn RR Selifonova OV Jessen HJ Steven JG Selmer T Buckel W 3-hydroxypropionic acid and other organic compounds Patent application 2001 no. PCT/US2001/043607

[B25] Jing X Meng X Xian M Biosynthetic pathways for 3-hydroxypropionic acid production Appl Microbiol Biotechnol 2009 82 995 1003 10.1007/s00253-009-1898-7 19221732

[B26] Csizmadia F JChem: Java applets and modules supporting chemical database handling from web browsers J Chem Inf Comput Sci 2000 40 323 324 10761134 10.1021/ci9902696

[B27] Brook A Kendrick D Meeraus A Raman R GAMS: A User's Guide GAMS Development Corporation 2002

[B28] Bixby RE Progress in linear programming ORSA J Computing 1994 6 15 22

[B29] Weiner H Enzymology and molecular biology of carbonyl metabolism 10 Gulf Professional Publishing 2001

[B30] Bitetti-Putzer R Joseph-McCarthy D Hogle JM Karplus M Functional group placement in protein binding sites: a comparison of GRID and MCSS J Comput Aided Mol Des 2001 15 935 960 10.1023/A:1014309222984 11918077

[B31] Varadwaj PK Lahiri T Functional group based ligand binding affinity scoring function at atomic environmental level Bioinformation 2009 3 268 274 19255647 10.6026/97320630003268 PMC2646862

[B32] Gasteiger J Engel T Handbook of Chemoinformatics 2003 Wiley-VCH

[B33] Holliday JD Hu CY Wilett P Grouping of coefficients for the calculation of inter-molecular similarity and dissimilarity using 2D fragment bit-strings Comb Chem High Throughput Screen 2002 5 155 166 11966424 10.2174/1386207024607338

[B34] Mavrovouniotis ML Group contributions for estimating standard gibbs energies of formation of biochemical compounds in aqueous solution Biotechnol Bioeng 1990 36 1070 1082 10.1002/bit.260361013 18595046

[B35] Mavrovouniotis ML Estimation of standard Gibbs energy changes of biotransformations J Biol Chem 1991 266 14440 14445 1860851

[B36] Deza MM Deza E Dictionary of distances 2006 Elsevier ISBN 0444520872

[B37] Croes D Couche F Wodak SJ Helden J Inferring meaningful pathways in weighted metabolic networks J Mol Biol 2006 356 222 236 10.1016/j.jmb.2005.09.079 16337962

[B38] Rion SC Teichmann SA Thoronton JM Homology, Pathway Distance and Chromosomal Localization of the Small Molecule Metabolism Enzymes in *Escherichia coli* J Mol Biol 2002 318 911 932 10.1016/S0022-2836(02)00140-7 12054833

[B39] Aguilar D Aviles FX Querol E Sternberg MJ Analysis of phenetic trees based on metabolic capabilities across the three domains of life J Mol Biol 2004 340 491 512 10.1016/j.jmb.2004.04.059 15210350

[B40] Pace NR A molecular view of microbial diversity and the biosphere Science 1997 276 734 740 10.1126/science.276.5313.734 9115194

[B41] Sankoff D Edit distance for genome comparison based on non-local operations Springer Berlin 2006

